# Recurrent Anti-NMDAR Encephalitis Necessitating Oophorectomy in an Adolescent Patient: A Case Report

**DOI:** 10.1155/2024/6150107

**Published:** 2024-10-15

**Authors:** Shadowen Caroline, Agrawal Nidhi, Fugina Alexa, Messersmith Cole, Terasaki Laurne, Allen Hannah, Goldberg Aaron, Pflugner Lindsey

**Affiliations:** ^1^Department of Obstetrics & Gynecology, Virginia Commonwealth University Hospital System, 1250 East Marshall Street, Richmond, Virginia 23298, USA; ^2^Internal Medicine, Family Health Centers at New York University Langone, 150 55th Street, Brooklyn, New York 11220, USA; ^3^Virginia Commonwealth University School of Medicine, 1201 East Marshall Street, Richmond, Virginia 23298, USA; ^4^ObGyn Associates of Advantia, 10750 Columbia Pike, Suite 700, Silver Spring, Maryland 20901, USA

## Abstract

**Background:** Anti-NMDA receptor (A-NMDAR) encephalitis is an autoimmune condition often associated with ovarian teratoma. Surgical removal of the teratoma is generally curative, and recurrence is uncommon.

**Case:** A 14-year-old female presented with psychiatric symptoms and was ultimately diagnosed with A-NMDAR encephalitis during a prolonged hospitalization. She was found to have bilateral ovarian teratomas, underwent laparoscopic bilateral ovarian cystectomy, and returned to neurologic baseline within 2 months. One year later, the patient was re-presented with similar symptoms and was diagnosed with recurrent A-NMDAR encephalitis. Initial imaging was negative for ovarian teratomas. After another prolonged hospitalization, repeat imaging ultimately demonstrated a suspected left ovarian teratoma. A left salpingo-oophorectomy was performed, and the patient's condition again fully recovered.

**Conclusion:** This case of A-NMDAR encephalitis presented with many atypical features including neuropsychiatric presenting symptoms, bilateral teratomas, and severe recurrence of disease. While imaging is the recommended modality for investigation of etiology, no teratoma was identified on the second presentation, leading to an ethical and clinical conundrum in this adolescent patient. More research is needed to investigate other diagnostic methods for A-NMDAR encephalitis without distinct teratoma on imaging in female patients.

**Summary**

• In female patients with A-NMDAR encephalitis, providers should maintain a high index of suspicion for ovarian teratoma even if initial imaging is negative for ovarian pathology, as teratoma and A-NMDAR encephalitis are often linked.
• Surgical management for patients with teratoma-associated A-NMDAR encephalitis is often curative and should be considered even for young patients of reproductive age in the case of refractory disease.
• Additional diagnostic methods for ovarian tumors should be explored, including advanced imaging and gonadal vein sampling.
• An adolescent patient experienced prolonged hospital admissions for anti-NMDA receptor encephalitis with recurrent ovarian teratomas (commonly associated with the disease) not immediately obvious on pelvic imaging, ultimately requiring unilateral oophorectomy.

## 1. Introduction

Anti-N-methyl-D-aspartate receptor (A-NMDAR) encephalitis is an immune-mediated neuroinflammatory disease characterized by antibodies against NMDA receptors in the hippocampus [1–3]. The disease typically presents with fever, malaise, and neurologic abnormality. It is often associated with neoplasm—most commonly for female patients, ovarian teratoma [4]. For cases of A-NMDAR encephalitis associated with teratoma, surgical removal of the teratoma typically resolves symptoms [4]. There are occasional reports of A-NMDAR encephalitis in the absence of obvious neoplasm on imaging, in which case it is often assumed that there is a microscopic neoplasm unidentified by standard imaging [5]. Even patients without visualized tumors typically respond to treatment with steroids and immunoglobulin [6].

Recurrence of A-NMDAR encephalitis is uncommon, with a rate of approximately 12% within 2 years of initial presentation [3]. For patients with teratoma-associated A-NMDAR encephalitis who undergo teratoma resection, recurrence is similarly uncommon and is typically associated with simultaneous teratoma recurrence [7, 8].

We encountered a 14-year-old patient whose initial episode of teratoma-associated A-NMDAR encephalitis resolved completely with surgical removal of ovarian teratoma, then 1 year later experienced a recurrence of encephalitis with no obvious teratoma on imaging evaluation. This case highlights multiple unusual features of A-NMDAR encephalitis and the longitudinal, multidisciplinary effort required for the care of such patients.

## 2. Case

A previously healthy 14-year-old gravida 0 female of South Asian ethnicity presented to a community hospital with malaise and acute-onset psychiatric symptoms including hyperactivity, abnormal comments and gestures, short-term memory loss, and visual hallucinations. She was diagnosed with a urinary tract infection and empirically started on oral cefalexin. Her psychiatric symptoms were thought to be related to discomfort from the infection, and she was discharged home. Five days after symptom onset, the patient experienced a witnessed 2-min episode of seizure-like activity including body stiffness, eye rolling, and salivation. This prompted her parents to re-present her to the emergency department. She was transferred to our academic tertiary care hospital for further workup.

On admission, the patient's neurologic exam revealed automatisms of bilateral hands, skin-picking, song-like noises, and intermittent loss of consciousness. The patient's parents reported no prior psychiatric or medical history, nor hospitalizations for the patient. Family history was significant for polycystic ovarian syndrome in her mother. Labs were significant for leukocytosis of 20.5, and the respiratory pathogen panel was positive for adenovirus by PCR. The basic metabolic panel was normal; urinalysis, urine drug screen, and thyroid function tests were normal. A computed tomography (CT) scan of the head demonstrated mild symmetric cerebral atrophy with preserved cerebellar volume. Magnetic resonance imaging (MRI) of the head revealed no acute intracranial abnormalities. An electroencephalogram (EEG) demonstrated seizure activity originating from the bilateral temporal lobes. She received one dose of intravenous fosphenytoin. A regimen of oxcarbazepine, acyclovir, and methylprednisolone was initiated for seizures and presumed encephalitis.

**Figure 1:**
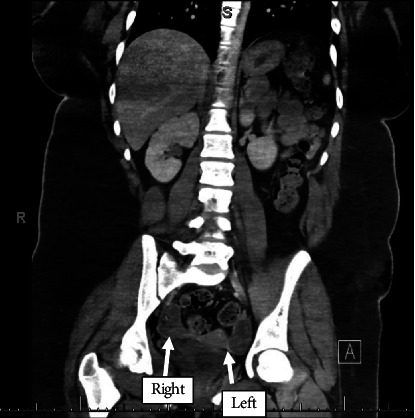
CT abdomen/pelvis demonstrating prominence of both ovaries including a small fat-containing area with punctate foci of adjacent hyperattenuation within the right ovary and an 8 cm^3^ ovoid area of relative hypoattenuation in the left ovary. Findings consistent with bilateral ovarian teratomas.

The patient underwent extensive infectious workup, including testing for Lyme disease, herpes simplex virus, Rocky Mountain spotted fever, ehrlichiosis, JC virus, and West Nile virus, all of which were negative. Lumbar puncture revealed cerebrospinal fluid (CSF) with normal glucose and protein but elevated total nucleated count (23 mm^3^), lymphocytic predominant. CSF was sent for NMDA-R antibodies with results pending. Abdominal CT revealed prominence of both ovaries including a small fat-containing area with punctate foci of adjacent hyperattenuation within the right ovary. Additionally, an ovoid area of relative hypoattenuation was noted in the left ovary measuring approximately 8 cm^3^, with central hyperattenuation (Figure 1), suggesting bilateral ovarian teratomas.

**Figure 2:**
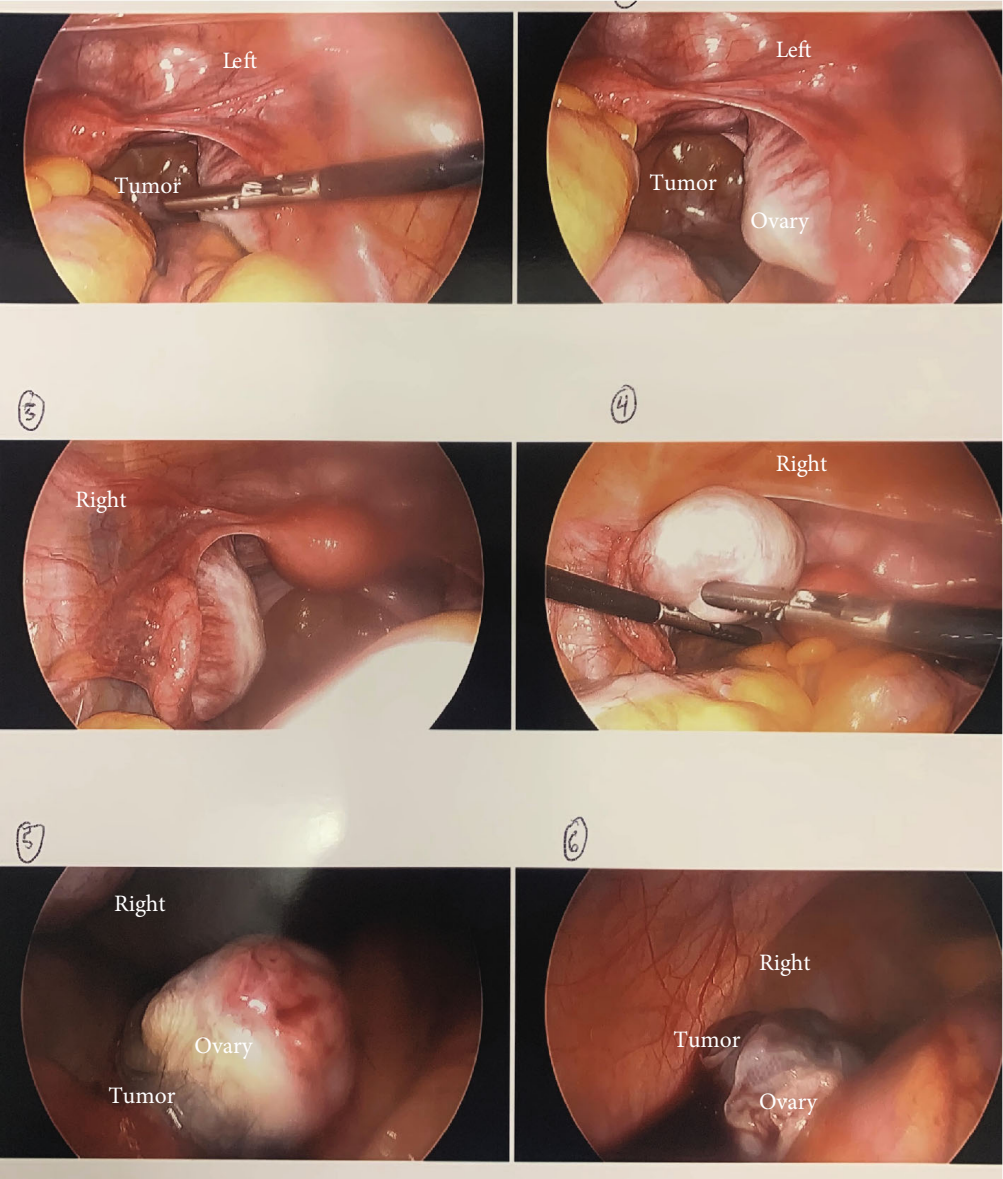
Intraoperative findings on laparoscopy demonstrating bilateral ovarian teratomas.

Additional pelvic imaging was considered but was deemed unlikely to affect the recommended treatment and thus not undertaken. After informed consent for ovarian cystectomy and possible oophorectomy, the patient underwent diagnostic laparoscopy. Intraoperatively, bilateral ovaries were noted to be enlarged with cystic regions suggestive of bilateral ovarian teratomas (Figure 2). Cystectomies were performed, and the remaining ovaries appeared normal. Both tumors were felt to be enucleated cleanly and completely at the time of surgery. Five days postsurgery, CSF resulted positive for A-NMDAR antibodies, and 2 days later, surgical pathology resulted in bilateral mature teratomas. The patient was started on a 5-day course of intravenous immunoglobulin (IVIG) treatment. Her neuropsychiatric symptoms markedly improved, and she was discharged home 6 days postoperatively. At a follow-up outpatient visit 2 weeks later, her neurologic status was 90% of baseline, though with ongoing amnesia from her hospitalization. Over the following year, all symptoms resolved with close neurologic follow-up.

**Figure 3:**
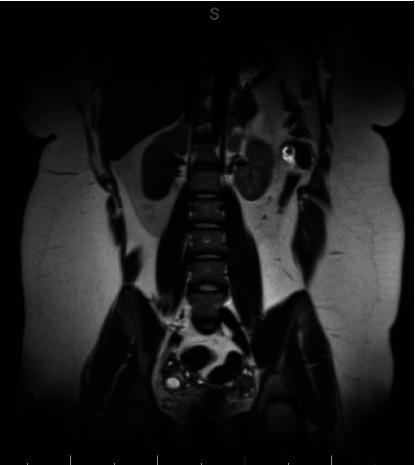
MRI abdomen/pelvis nondiagnostic for teratoma.

Fourteen months after initial discharge, the patient re-presented to our Emergency Department with headache, memory loss, involuntary body movements, and an altered speech pattern, a constellation of symptoms nearly identical to that for which she had initially presented over a year prior. The patient was admitted and underwent lumbar puncture, again revealing NMDAR antibodies at a higher level than during her previous admission, confirming the recurrence of A-NMDAR encephalitis. Head MRI showed no abnormalities. Pelvic ultrasound and MRI (with image slice width at a standard 3.0 mm) were both negative for teratomas (Figure 3). The patient was started on methylprednisolone, followed by IVIG.

Despite intensive inpatient treatment and multidisciplinary collaboration over the subsequent days, the patient's symptoms did not improve. She continued to experience severe agitation, insomnia, and confusion and exhibited increasingly bizarre behaviors. EEG confirmed seizures, and a regimen of levetiracetam, lacosamide, and rituximab was initiated. Gynecology was again consulted, but given normal imaging studies, surgical management was deferred.

**Figure 4:**
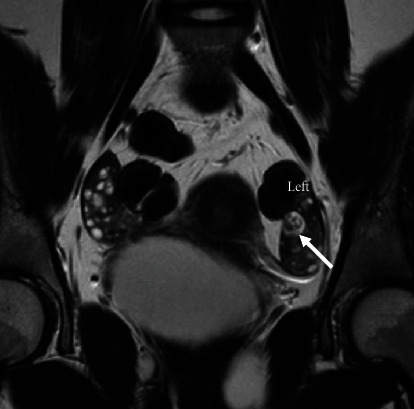
Repeat MRI abdomen/pelvis demonstrating 1.2 × 1.0 × 1.2 cm complex structure with macroscopic fat in the left ovary concerning for recurrent unilateral teratoma.

**Figure 5:**
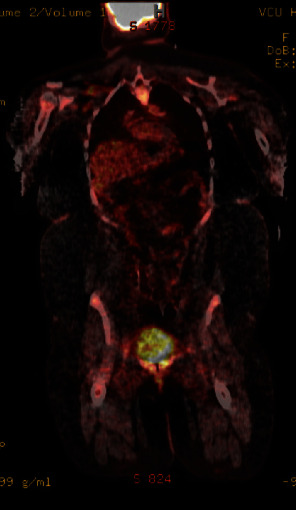
PET scan nonrevealing.

Continued neuropsychiatric decline was ongoing despite medical treatment. Much debate was held amongst the gynecology service about how to proceed given no obvious teratoma but possible presence given the recurrence of A-NMDA encephalitis. In particular, the patient's parents strongly desired ongoing gynecologic evaluation and treatment. Five weeks after initial imaging, a repeat pelvic MRI was performed and now demonstrated a 1.2 × 1.0 × 1.2 cm complex structure with macroscopic fat in the left ovary concerning recurrent unilateral teratoma (Figure 4). A PET scan of the chest, abdomen, and pelvis was ordered, given that PET has been known to identify immature teratomas that fail to present on traditional imaging [9]; however, this was negative (Figure 5). Serum alpha-fetoprotein (AFP) was also ordered, as this tumor marker is often elevated in immature teratomas [10]; the patient's AFP level was elevated to 18.2.

Informed consent was obtained for diagnostic laparoscopy and likely unilateral oophorectomy. This consent process included multiple multidisciplinary conversations with the patient's parents given the morbidity of oophorectomy in an adolescent patient. Again, there was much debate amongst the institution's gynecologists regarding how aggressively to treat, especially in the case of negative intraoperative findings, given the age and reproductive potential of the patient. Ultimately, intraoperatively, both ovaries appeared normal—unlike the obviously abnormal intraoperative findings of the first surgery—but given the repeat MRI and the prolonged severe derangement of the patient's neurologic status, the decision was made to remove the left ovary.

Postoperatively, symptoms improved, AFP decreased, and the course of rituximab was completed. Unfortunately, due to a lab error, the left ovarian specimen was not processed and a final pathologic diagnosis was not achieved. The patient was discharged home 1 week postoperative with a plan for completion of IVIG and steroid taper as an outpatient. She was continued on trazodone, melatonin, lacosamine, and clonidine. At 2 weeks postoperative, she had returned to 75% of her neuropsychiatric baseline. At 3 months postoperative, she had returned to 100% of her neuropsychiatric baseline. A plan was made to receive rituximab every 6 months as well as surveillance pelvic MRI.

## 3. Comment

Our patient's case is notable for several reasons. First, our patient presented both times with psychiatric symptoms and seizures. Patients with teratoma-associated A-NMDAR encephalitis typically present with fever and decreased consciousness; psychiatric symptoms are classically seen later in the clinical course [7, 8]. Also, ovarian teratomas associated with A-NMDAR encephalitis are most commonly unilateral; in a case series by Dalmau et al. including 98 patients with A-NMDAR encephalitis [2], 53 patients were found to have ovarian teratomas, but only eight were bilateral. However, our patient had bilateral teratomas during her first admission. Additionally, recurrence of A-NMDAR encephalitis is uncommon, with most patients experiencing a full recovery, unlike our patient [3, 7, 11]. Further, initial imaging upon admission for our patient's recurrence of anti-NMDAR encephalitis did not show evidence of ovarian teratoma and represented a clinical dilemma on how aggressively to treat based on history and suspected presence of teratoma.

Recurrence of A-NMDAR encephalitis without recurrent teratoma is uncommon. During our patient's second admission, pelvic imaging was initially negative for teratoma. Given her protracted course, gynecologists at our institution debated how aggressively to treat, based on the assumption that microscopic teratoma could be present—enough to trigger the formation of antibodies but too small to visualize on gross imaging. Initially, the gynecology team did not have a role to intervene. However, due to the patient's protracted course and increasing morbidity, a repeat pelvic MRI was obtained 5 weeks later and did demonstrate a tumor. It remains unclear why the initial imaging was negative for teratoma; it could have been that the MRI slice thickness was not narrow enough to capture subtle pathologic changes (though the standard slice width of 3.0 mm at our hospital is standard for pelvic imaging), or the tumor recurrence could not have been large enough to visualize on imaging yet. Either way, this prolonged period in which the patient's clinical condition was worsening despite negative imaging was difficult both for her family and her medical teams. Another case report by Walker et al. describes the case of a 14-year-old female admitted to the ICU for over 6 months with A-NMDAR encephalitis with imaging negative for teratoma [12]. Walker et al.'s patient slowly improved, was discharged, and then had symptom recurrence months later; repeat imaging at the time of her re-presentation revealed ovarian teratoma absent on initial imaging. Like in our case, the removal of the teratoma in Walker et al.'s patient was curative. Both cases highlight the importance of maintaining a high index of suspicion for ovarian teratoma in female patients with A-NMDAR encephalitis even if initial imaging is negative for ovarian pathology and to consider surgical management in spite of negative imaging if the clinical scenario warrants.

Every effort was made by this adolescent female to achieve a diagnosis prior to surgical intervention, given the goal of retaining her ovaries. When initial imaging on re-presentation was negative for teratoma, there was thought to be no role for gynecologic intervention. However, because the patient's course was so severe and protracted, the family advocated quite strongly for ongoing gynecologic workup and intervention, which prompted the AFP and PET scan. Fortunately, the repeat MRI was positive. Our team did discuss the MRI reading (both the initial and second MRIs) with the radiologist and verified that they did not note any abnormal changes whatsoever on the right ovary, but they felt confident that the left ovary did note changes characteristic of ovarian teratoma. Intraoperatively, our team performed a thorough visual inspection of both ovaries and did not note any gross abnormalities in either one. However, given the MRI results, the patient's severe and intractable case, and the patient's family's desires, we moved forward with an oophorectomy. One idea that could be considered in the future would be sampling of the gonadal veins for anti-NMDAR antibodies, with the goal of both uncovering confirmatory positive antibodies in the blood supply around the ovary and laterality to a suspected microscopic teratoma. Ovarian vein sampling for testosterone has been used for suspected androgen-secreting tumors to identify which ovary may contain microscopic tumors not obvious on imaging [13]. In cases of A-NMDAR encephalitis without obvious teratoma, it is possible that gonadal vein sampling for antibodies could be of utility to determine laterality. Further studies are needed to evaluate this as a diagnostic tool.

This case of a patient with recurrent bilateral A-NMDAR encephalitis without teratoma on initial imaging provides teaching points and ideas for future methods of diagnosis and treatment. For patients with this disease, early treatment with immunotherapy, multidisciplinary supportive care, and resection of surgical teratoma if present are all critical parts of management.

## Data Availability

The data that support the findings of this study are available from the corresponding author upon reasonable request.
